# Diseases of the Ear

**Published:** 1912-06

**Authors:** 


**Diseases of the Ear.** By W. Milligan, M.D., and Wyatt Wingrave, M.D. Pp. xxvii, 596. London: Macmillan and Co. Limited. 1911.

Chapters I to III, on the development, anatomy of the external ear, and physiology of the sound-conducting apparatus, occupy the first seventeen pages. The anatomy of the internal ear is separately described in Chapter XXXII, but the forty-three pages on the examination of the ear, etc., is throughout excellent, and the various tests are clearly described, and their value contrasted. Separate chapters are devoted to deformities of the external ear, injuries, diseases of the auricle, foreign bodies, diseases of the external auditory meatus, of the middle ear, the membranal inflammations, traumatic lesions, exudative catarrh, chronic adhesive catarrh, acute suppurative inflammation of the middle ear, epitympanic suppuration, chronic suppurative inflammation of the middle ear, and so forth, and all are discussed thoroughly. In labyrinthine affections Dr. Milligan, as one of the pioneers in the modern developments of operative treatment of these regions, might be expected to reach a high standard of excellence, and in this we are not disappointed.

As a scientific and practical guide to modern otology this text-book is unsurpassed. Every section is treated with a masterly clearness and authority, and nothing of importance is omitted. Dr. Milligan has been ably supported by the pathological contributions and illustrations of Dr. Wyatt

Wingrave, who has done so much to advance our knowledge of the pathology of the ear, nose and throat. Richly illustrated in colour and in black and white, well written, authoritative, concise and complete, the work is a credit to British otology.

